# A Benchmark Arabic Dataset for Arabic Question Classification using AAFAQ Framework

**DOI:** 10.1038/s41597-025-05688-0

**Published:** 2025-08-18

**Authors:** Mariam Essam Abdelaziz, Mohanad A. Deif, Shabbab Ali Algamdi, Rania Elgohary

**Affiliations:** 1 Department of Computer Science, College of Information Technology, Misr University for Science and Technology (MUST), P.O. Box 77, Giza, Egypt; 2 Research Institute of Sciences and Engineering, University of Sharjah, Sharjah, United Arab Emirates; 3 Department of Software Engineering, College of Computer Science and Engineering, Prince Sattam bin Abdulaziz University, Al Kharj, Saudi Arabia; 4 Faculty of Computer and Information Sciences, Ain Shams University, Cairo, Egypt

**Keywords:** Computer science, Databases

## Abstract

Arabic Natural Language Processing (NLP) is still faced with the complexity of the language’s morphology and the limited availability of quality annotated resources. In this paper, we introduce an open-domain dataset of 5,009 Modern Standard Arabic (MSA) questions labeled according to AAFAQ framework that has11 linguistic and cognitive aspects, e.g., Question Particle, Question Particle Type, Intent, Answer Type, Cognitive Level, and Temporal Context. Based on the AAFAQ Framework (Arabic Analytical Framework for Advanced Questions), the dataset is designed to support semantic and cognitive understanding for Arabic Question Classification and related tasks. The dataset’s effectiveness was validated by fine-tuning state-of-the-art models. AraBERT achieved 100% accuracy on Question Particle Type classification and 94.95% on Intent classification. Integration within a generative question-answering system with Alpaca + Gemma-9B Unsloth improved evaluation metrics, including BLEU (+37.6%), ROUGE-1 (+132%), and BERTScore (+17.3%), validating the dataset’s value in both classification and generation tasks. Despite its broad coverage, the dataset includes underrepresented categories, e.g., Sociology and Volunteering, to be considered in future extensions. AAFAQ is a foundation benchmark for the advancement of Arabic question comprehension, with prospective applications in education, cognitive computing, and multilingual AI system creation.

## Background & Summary

Arabic, among the most spoken languages in the world, represents an interesting case study for NLP due to its rich morphology, diverse syntactic structures, and diglossia, with MSA coexisting with regional dialects. Although NLP has gone through a remarkable development over the past decade, Arabic is still not well-represented by qualitative or quantitative annotated datasets adapted to specific tasks like Question-Answering (QA) or classification^1–4^.

Question Answering System (QAS) came into being in the information age and became one of the basic enactments in the field of NLP, which grew the users’ interaction with technology. These types of systems, which were designed to interpret natural language and return exact answers to whatever question a given user may have at a particular point in time, have recently drastically changed the face of search engines, virtual assistants, and automated customer support^5–7^. They allow access to information very easily; hence, the Question Answering Systems (QASs) have considerably improved human-machine interaction for a great variety of domains. QASs assumes a pivotal importance in the more recent trends and developments of NLP, offering the ability of automatic information retrieval and answering user inquiries in natural languages. Quite recently, a host of QASs have targeted Arabic for domain-specific languages, including health, education, and Quranic applications^8^. Most resource materials are not strong enough and universally applicable to deal with a wider application-oriented question analysis, especially classification issues^9^. This gap therefore calls for domain-specific open-domain datasets which could be helpful in the development of robust and sophisticated QAS for Arabic^4,8^.

Question classification is considered to be one of the most basic factors of any good QAS, as it determines the expected type of answer. The process will narrow the search space and improve the relevant information retrieval process^7,10^. In Arabic QAS, correct classification is seriously vital on account of the syntactic and morphological variability of the language. Current methods using state-of-the-art vary from deep learning-based classifiers to traditional methods such as modified TF-IDF, with varied degrees of success^11,12^. Most, in fact, classify questions into four categories, namely factual, non-factoid, list, or yes/no questions, often leveraging taxonomies such as Li and Roth-which were not optimally suited to address Arabic’s distinctive linguistic and structural challenges^1^. This is because the repeated and generalized classifications limit the ability of these systems to capture finer details of user queries for interpretation and response through a deeper understanding of meaning^13,14^.

To cover these gaps, we present the AAFAQ Framework (Arabic Analytical Framework for Advanced Questions) and its benchmark dataset. AAFAQ Framework is a novel suggested modular and extensible framework that aims to provide multi-layer Arabic question linguistic analysis. It espouses a systematic method for taking into consideration the semantic, cognitive, and context dimensions of Arabic questions to facilitate enhanced classification, comprehension, and reasoning for QAS. The suggested title AAFAQ (آفاق), which in Arabic means horizons, echoes the vision of advancing the horizons of Arabic question comprehension and the enhancement of advanced NLP applications.

This paper is committed to presenting the dataset that supports AAFAQ framework that is an open-domain benchmark of 5,009 carefully annotated Arabic questions. The dataset follows the standards of the AAFAQ Framework in providing annotations on various dimensions, including Question Particle, Intent, Answer Type, Cognitive Level, Temporal Context, and others. It attempts to bridge the shortage of Arabic NLP resources by enabling researchers and developers to build more precise and context-aware Arabic QAS models.

**Fig. 1:**
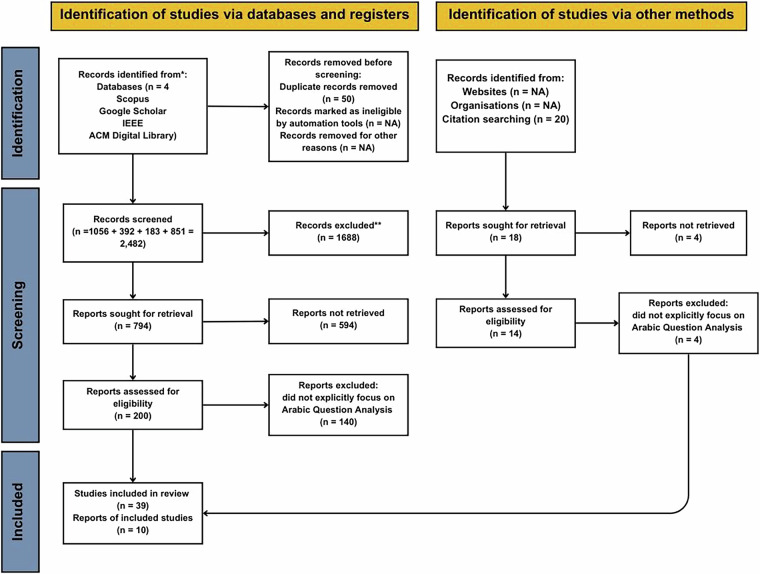
Prisma Chart for Sematic representation of the study design and article selection process for the development of the dataset.

It was created with a systematic and extensive methodology using well-defined inclusion and exclusion criteria, and an exhaustive search in Arabic QAS research for the period between 1993 and 2024. Our final list consisted of a total of 49 highly relevant studies, which informed us about the development of the dataset and annotation system. Figure 1 illustrates the PRISMA diagram outlining the article selection process. With its rich annotations and alignment to the AAFAQ Framework, the dataset offers a valuable contribution to advancing research in Arabic NLP, QAS, and educational and cognitive applications. The dataset not only advantages Arabic applications but can also inspire the creation of analogous multi-layer classification schemes for other languages.

The selection criteria, therefore, put much emphasis on the selection of those publications that, in addition to addressing Arabic QAS question analysis and classification, define comprehensive methodologies, tools, or techniques used in the construction of such systems. Exclusions have been made where studies had a sole focus on community-based QAS, did not indicate explicit methodologies in question analysis, or were totally unrelated to the broader area of Arabic NLP question classification.

Information sources included Scopus, IEEE, and the ACM Digital Library, among other large academic databases, and search engines including Google Scholar. The search strategy involved Boolean operators and keywords. Examples of search terms include “Arabic Question Answering System” OR “Arabic Question Analysis” AND “Dataset” OR “Tools” OR “Survey” OR “Resources,” and other related phrases tied to the main subject indexes “Natural Language Processing,” “Text Mining,” “Information Retrieval,” and “Computational Linguistics.” Searches were made from 1993 up until 2024 to capture a broad, inclusive review of the available literature.

Article selection was done in a multi-stage approach. Of the selected databases, 2,482 papers were identified; after removing duplicates and irrelevant studies, 200 papers were shortlisted on abstracts and titles, which, after full-text review, resulted in a final selection of 49 studies directly relevant to methodologies in Arabic QAS question analysis and classification. Indeed, this is quite an elaborate process; it is visually depicted in Fig. 1, which schematically represents how the study has been designed.

This structured and detailed approach shows how much effort went into developing the dataset to make it applicable and relevant for the furtherance of Arabic NLP research and QAS methodologies.

Although they follow the first stage of the question analysis in this domain, most of the time it is not visible that specialized datasets exist. What is the best description of it: Although many datasets exist for Arabic QAS, not all of them are created with question analysis in mind. Other specialist resources are thus still needed in this domain. Some researchers only follow the development of specialized datasets in this approach line. Some of the good examples are the development in the work of Li and Roth^1,15^. Note that there are no Arabic language datasets created specifically for this task. The performance and effectiveness of QAS rely on question analysis and classification; thus, this gap is so critical. As the number of studies and resources available grows in the field of Arabic NLP, one realizes that the current phase of scantily focused datasets is totally irrelevant to the newer, more evolved Arabic QAS being created. Therefore, this addresses the gap as a considerable area for future consideration in research and development. There is, no doubt, still a considerable need in this respect for tailored datasets to meet the complexities and specificities of question analysis and classification in Arabic. Such data sets, as they get created and made available, will not only bring the research concerned closer and more integrated but will take the overall effectiveness and capabilities of Arabic QAS to a whole new level^8^.

This dataset fills the crucial gap in Arabic NLP with a rich resource for Arabic question classification, allowing for unprecedented progress in education, cognitive research, and more Arabic-specific NLP applications. Although the dataset is in Arabic, the underlying taxonomy in AAFAQ can easily be adapted to non-Arabic applications, since the semantic and cognitive dimensions that are covered by question classification are universal. The topic is related to NLP and AI in question classification, semantic analysis, and the training of question-answering systems. In education, this dataset allows for intelligent automation of some tutoring systems and improves cognitive skill assessments. Due to its open-domain nature, the usability spans through applications in different domains, from health care to technology, up to public services; this is going to be an essential base resource for innovation for Arabic NLP and beyond.

### Related work

In recent years, QAS has been receiving a lot of interest in the Arabic NLP community as a result of the expanding demand for intelligent, language-specific applications like intelligent tutors, search engines, and information retrieval systems. In spite of the progress in NLP, Arabic lacks sufficient quality annotated data, even more so for question classification. This is mainly because the language presents special challenges in the form of rich morphology, syntactic variation, and diglossia between Modern Standard Arabic and regional dialects.

A number of datasets have been created to enable Arabic QA. Some of the earlier efforts include the dataset of DAWQAS^16^, which has around 3,200 annotated Arabic “why” questions with a causal relation. While DAWQAS is a useful dataset for causal QA, it only covers one question type and does not consider broader semantic or cognitive categorizations.

The WikiQAar dataset^17^ holds some 3,000 open-domain Arabic questions for use in answer selection tasks. Likewise, the XQUAD Arabic subset^18^ includes 1,190 span-based questions derived from SQuAD for multilingual Question-Answering (QA). Both are more suited for extractive QA than question classification. In addition, the Quran-QA dataset^19^ represents a dedicated domain-specific resource based on Quran text, with some 1,500 to 2,000 questions. While this will be useful for religious and theological use, it’s limited in use to a single domain and does not have general-purpose annotations.

Another dataset pertinent thereto is the So2al-wa-Gawab dataset^20^, which includes 10,000 Arabic question-answer pairs taken from Wikipedia. While it is among the largest provided Arabic QA datasets, it is annotated for span-based answer retrieval but lacks question categorization.

Most such datasets are used for answer retrieval or extraction and lack explicit labels for question type, intent, or cognition. All the classification done so far tends to be restricted to basic taxonomies, i. e., factoid/non-factoid classification, or taxonomy-based answer type labeling as in Li and Roth (2002)^9^. Some recent approaches, e.g., those by Hamza *et al*. (2022)^1^ and Balla *et al*.^11^, have used deep learning models for Arabic question classification. Still, these works tend to make use of limited/task-specific datasets, which limit the generalizability and reliability of the models.

In contrast to previous work, the proposed a Dataset aims to fill this gap by providing a benchmark, open-domain Arabic dataset specifically designed for multi-dimensional question classification. AAFAQ introduces a rich annotation framework that extends beyond basic question types to include 11 semantic and cognitive dimensions: Question Particle, Particle Type, Question Type, List, Answer Type, Intent, Cognitive Level, Subjectivity, Temporal Context, and Purpose Context. To the best of our knowledge, this is the first dataset of its kind in the Arabic NLP literature that supports fine-grained, multi-label question classification, making it a valuable resource for advancing Arabic question understanding and improving the development of Arabic QAS.

To facilitate comparison, Table 1 presents a comparative overview of existing Arabic QA datasets versus the proposed dataset.

**Table 1:** Comparative Overview of AAFAQ and Existing Arabic QA Datasets.

Dataset	Size	Task Type	Domain	Annotation Type	Multi-dimensional Question Classification
**DAWQAS**	~3,200	Answer Selection	“Why” Questions	Question-Answer Pairs with causal labels	✗
**WikiQAar**	~3,000	Answer Selection	Open-domain	Question-Answer Sentence Pairs	✗
**XQUAD (Arabic)**	1,190	Span Extraction	Open-domain	SQuAD-style answer spans	✗
**Quran-QA**	~1,500–2,000	Answer Retrieval	Quranic Domain	Domain-specific Q/A pairs	✗
**So2al-wa-Gawab**	10,000	Answer Retrieval	Open-domain (Wikipedia)	Span-based Q/A without classification	✗
**AAFAQ (Proposed)**	**5,009**	**Question Classification**	**Open-domain**	**9-dimensional annotations** (e.g., Intent, Cognitive Level, Answer Type, Purpose Context, etc.)	✓

## Methods

### Data collection

The development of the dataset involved a lengthy process of gathering different Arabic questions from different open and publicly available sources. The data collection first was undertaken using manual collection and exploiting existing datasets. The multi-source strategy enriched the dataset to offer a wide variety of question types, domains, and contexts to support Arabic NLP research. Several open-domain Arabic question-answering datasets were incorporated into the dataset:

- Why Questions Dataset (DAWQAS): Available on GitHub, this dataset focuses on Arabic “Why” questions and was used to supplement causal question types in our dataset^16,21^.
- WikiQAar: A Hugging Face dataset, created for open-domain Arabic QA^17,22^.
- XQUAD Dataset: A multilingual SQuAD dataset, publicly available on Github^18,23^.
- Quran-QA Dataset: A Quranic text domain-specific question-answering dataset that can be downloaded from Git Lab^19,24,25^.

All these datasets are released under permissive licenses and were reused in compliance with their terms and conditions. This step provided a richer diversity of question styles, linguistic expressions, and topical coverage. Only data available without restrictions or copyright limitations were considered.

## Data Records

The dataset supporting this study is publicly hosted on the Dryad Digital Repository and can be accessed at^26^. It comprises structured files that represent the curated benchmark resource developed for Arabic question classification under the AAFQ taxonomy.The dataset package includes the following files:

- **AAFAQ_Dataset.csv (2.82 MB):**
  This primary file contains the labeled question instances used in classification experiments. Each record includes fields such as question text, corresponding category label(s), and possibly metadata relevant to linguistic or semantic features. The structure is provided in a flat CSV format with UTF-8 encoding.
  This is a Comma Separated Values file containing 15 columns and 5,009 rows. Each question is uniquely represented as a row in the dataset. Supplementary Table^1^ describes each column along with its attribute.
- **README.md (3.06 KB):**

This accompanying file offers documentation detailing the schema of the dataset, annotation process, taxonomy explanation, and recommended usage protocols. It serves as a guideline for users seeking to replicate experiments or integrate the dataset into new natural language processing pipelines.

The dataset is published under a public domain dedication, ensuring unrestricted reuse with proper citation. These records are intended to support reproducibility, encourage comparative benchmarking, and facilitate downstream research in Arabic NLP and intelligent question classification system.

The proposed dataset underwent a thorough validation process to ensure its reliability, quality, and utility for Arabic NLP tasks. The validation covered three main aspects: (1) dataset construction, (2) annotation consistency, and (3) experimental evaluation for question classification and answer generation tasks.

### Dataset construction

**Fig. 2:**
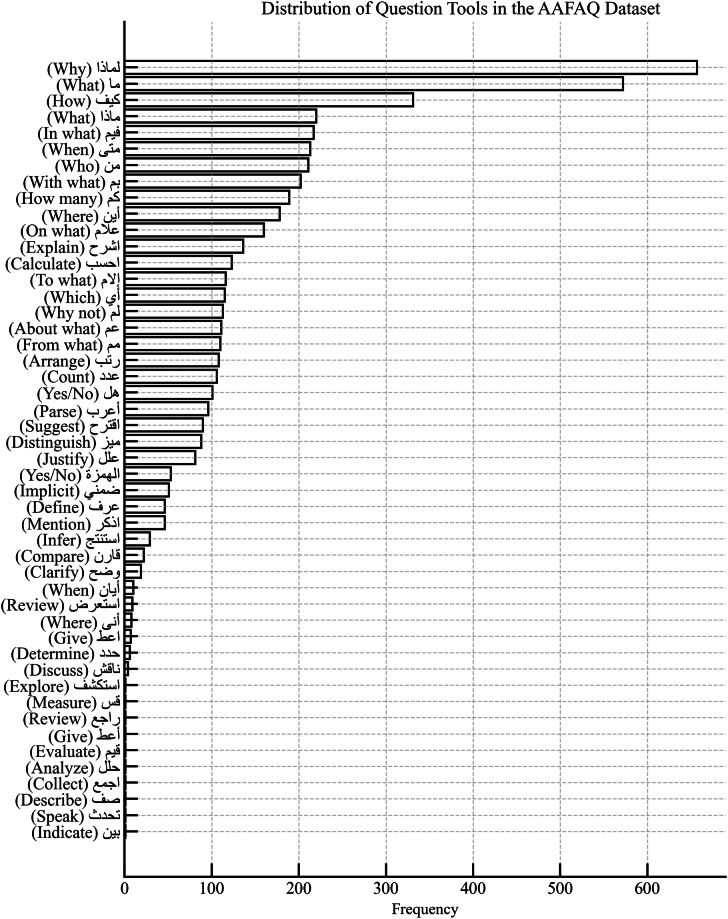
Frequency Distribution of Question Particles in the Dataset.

The proposed dataset provides a rich variety of distributions across its annotations, offering insights into Arabic question patterns and classifications. Key distributions are shown in Fig. 2.

**Fig. 3:**
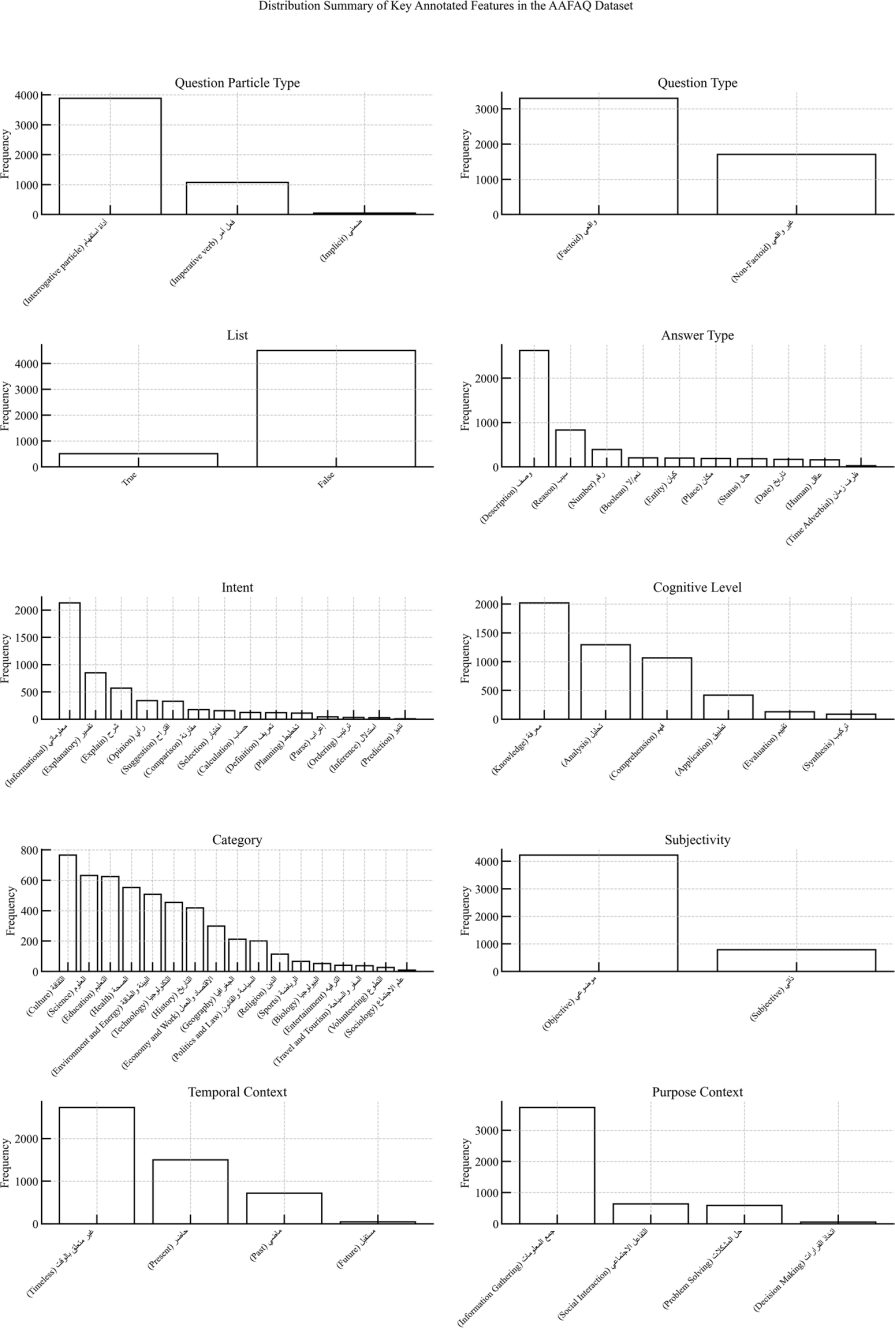
Distribution of Annotated Linguistic and Cognitive Features in the AAFAQ Dataset.

To assess the internal composition of the dataset, a comprehensive analysis of feature distributions was conducted across all annotated dimensions as illustrated in Fig. 3:

- **Question Particle and Particle Type:** The most frequent interrogative forms were “لماذا” (Why), “ما” (What), and “كيف” (How), indicating a strong presence of causal and descriptive queries. The majority of questions utilized explicit interrogative particles, followed by imperative verbs and a smaller subset employing implicit formulations.
- **Question Type:** The dataset shows a predominance of factoid questions (واقعي), accounting for approximately 66% of the total, while non-factoid entries (غير واقعي), which involve reasoning or explanation, constitute the remaining 34%.
- **List Feature:** The majority of the dataset (89.7%) comprises questions that expect singular responses, while only 10.3% require list-based answers, indicating its suitability for both single-answer and multi-response QA systems.
- **Answer Type:** Descriptive answers were the most common (2,621 entries), followed by numerical, temporal, and causal types. Boolean and spatial responses were among the least frequent, illustrating a wide spectrum of expected answer forms.
- **Intent:** The dataset is largely driven by informational intent (2,132 questions), followed by explanatory, comparative, and opinion-based intents. Predictive and planning-oriented questions were underrepresented, highlighting areas for potential future enrichment.
- **Cognitive Level:** Analysis revealed an emphasis on foundational cognitive skills, particularly Knowledge (2,021 entries) and Comprehension. Higher-order levels such as Synthesis and Evaluation were present but less frequent, aligning with the dataset’s orientation toward analytic and educational tasks.
- **Topical Category:** The dataset spans a diverse array of domains. The most represented categories include Culture (765), Science (632), Education (624), and Health (552). Conversely, underrepresented domains such as Sociology (9 entries) and Volunteering (26 entries) highlight opportunities for targeted expansion.
- **Subjectivity:** Objective questions dominate the dataset (4,221 entries), reinforcing its alignment with tasks that require factual and verifiable answers. Subjective questions, while fewer in number, provide a complementary semantic layer for evaluating evaluative and opinion-based comprehension.
- **Temporal Context:** Most questions were classified as timeless (2,728 entries) or related to the present (1,499 entries), with minimal representation of past or future contexts. This distribution indicates the dataset’s predominant focus on general knowledge and current-event applicability.
- **Purpose Context:** The leading communicative purpose was information gathering (3,729 questions), followed by social interaction, problem solving, and decision-making. Predictive purposes were rare, indicating an area for further diversification.

### Correlation analysis across dimensions of annotation

**Fig. 4:**
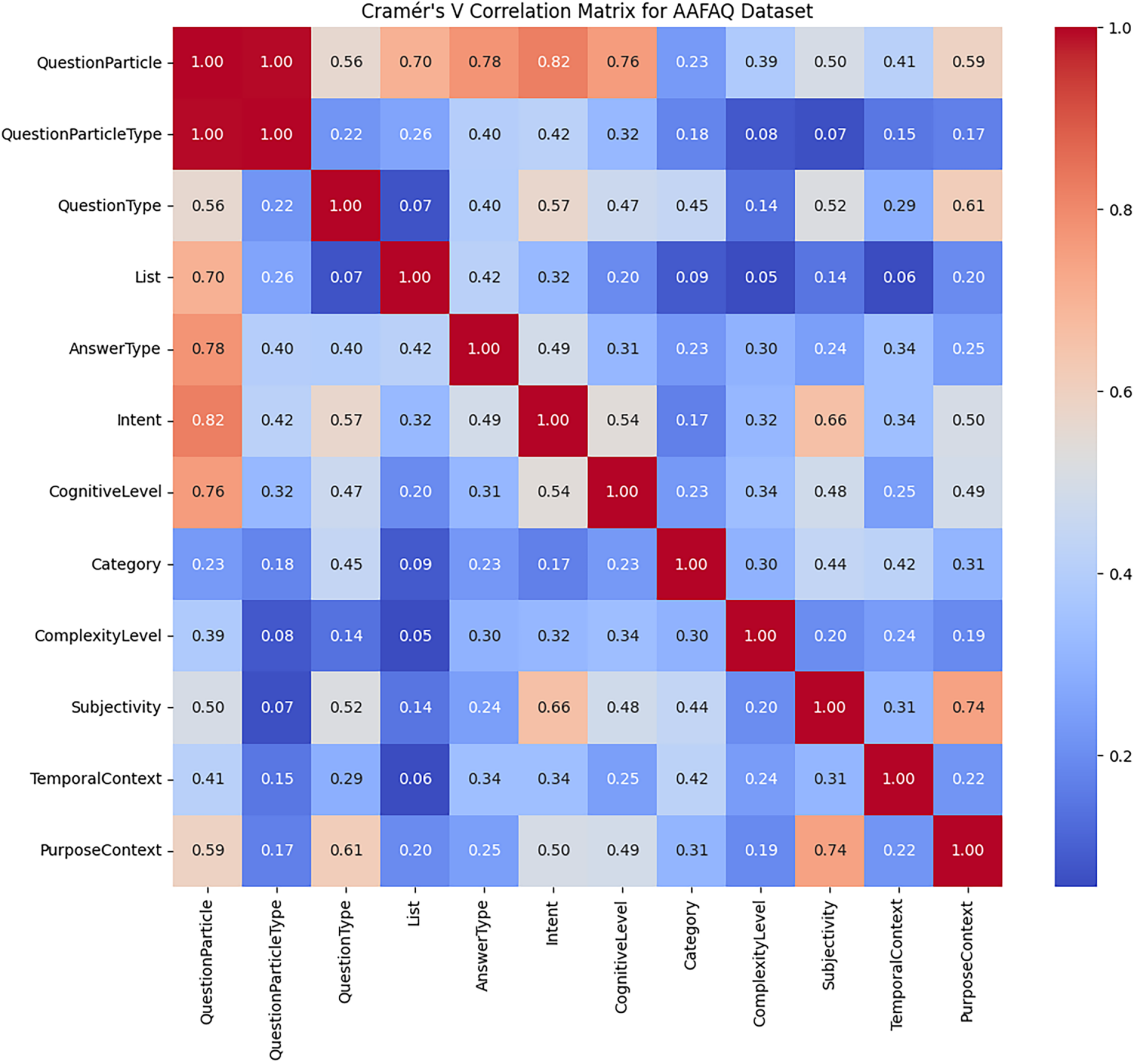
Correlation Matrix between Features Dimensions.

In addition to the convergence validation of the proposed dataset internal structure, we performed Cramér’s V correlation analysis^27^ as a measure of the associations’ strength between the categorical features in the proposed dataset. In the resulting values, as presented in Table 2 and illustrated in Fig. 4, the insightful interdependencies among the annotation dimensions are shown.

**Table 2:** Overview of The Dataset Features, Types, and Categories.

Strength	Feature 1	Feature 2	Correlation	Interpretation
**Strong**	QuestionParticle	QuestionParticleType	1.00	Perfectly associated, which is expected, because QuestionParticleType is likely derived directly from QuestionParticle.
**Strong**	QuestionParticle	Intent	0.82	Question particles are strongly indicative of the intent behind the question.
**Strong**	QuestionParticle	AnswerType	0.78	The question particle has a strong role in determining the expected answer type.
**Strong**	QuestionParticle	List	0.70	Certain question particles are heavily associated with whether the answer is in a list form.
**Strong**	Subjectivity	PurposeContext	0.74	The subjective or objective nature of the question is highly related to its purpose.
**Strong**	Intent	Subjectivity	0.66	The intent influences whether a question is subjective or objective.
**Moderate**	QuestionType	PurposeContext	0.61	Different question types tend to serve different purposes.
**Moderate**	QuestionType	Subjectivity	0.52	Certain question types tend to be more subjective or objective.
**Moderate**	Intent	QuestionType	0.57	The question type is moderately dependent on the intent.
**Moderate**	QuestionType	AnswerType	0.57	The answer type is moderately determined by the type of the question.
**Moderate**	QuestionParticle	CognitiveLevel	0.76	Some particles might lead to cognitively harder or easier questions.
**Moderate**	CognitiveLevel	Intent	0.54	Higher cognitive levels are related to certain question intents.
**Moderate**	Subjectivity	CognitiveLevel	0.48	Harder questions may tend to be more or less subjective.
**Moderate**	Subjectivity	ComplexityLevel	0.44	Complexity contributes to subjectivity.
**Moderate**	ComplexityLevel	CognitiveLevel	0.34	Complexity and cognitive level are related but not strongly.
**Moderate**	PurposeContext	CognitiveLevel	0.49	The purpose affects the cognitive level of the question.
**Weak**	Subjectivity	ComplexityLevel	0.44	—
**Weak**	TemporalContext	Subjectivity	0.31	—
**Weak**	PurposeContext	AnswerType	0.25	—
**Weak**	ComplexityLevel	Intent	0.32	—

Interestingly, the Question Particle feature holds a strong correlation with Question Particle Type (Cramér’s V = 1.00) by virtue of the taxonomy’s design. It indicates strong correlations with Intent (0.82), Answer Type (0.78), and List (0.70) as well, as interrogative words play a significant determining role in the interrogative nature and the cognitive and semantic characteristics of questions.

In the same way, Subjectivity correlates foremost with Purpose Context (0.74) and secondarily with Intent (0.66) and Cognitive Level (0.48), validating the senses in which subjective and objective questions differ significantly in purpose and difficulty.

Dimensions such as Cognitive Level, Question Type, and Purpose Context correlate moderately but importantly with one another (0.49 to 0.57 by Cramér’s V), which indicates that different question types require different extents of cognitive processing and have disparate communicative intentions.

These findings confirm the expressiveness and coherence of the AAFAQ schema annotation, substantiating the interplay between linguistic, semantic, and cognitive aspects among Arabic queries.

### Annotation consistency

The annotation process was carefully designed to be of high reliability and consistency. Manual annotation was performed by seasoned annotators following a structured guideline adapted from the AAFAQ Framework. To measure annotation consistency, an independent annotation of a random 10% subset of the data yielded an inter-annotator agreement (IAA) score of 85%, reflecting high reliability of annotation. Blinding protocols were observed to prevent possible bias by masking contextual information and question sources during annotation. Several rounds of quality assurance were also conducted, and approximately 1,500 questions were removed due to duplication, irrelevance, or inconsistency. The final dataset consists of 5,009 rigorously curated questions with complete annotations along the 11 dimensions of the AAFAQ Framework.

### Experimental evaluation

In order to continue verifying the validity of the dataset, thorough experimental testing was performed. The dataset was used in a multi-label classification task over nine main features: Question Particle Type, Question Type, List, Answer Type, Intent, Cognitive Level, Subjectivity, Temporal Context, and Purpose Context. Several models were trained and tested, namely RNN-based models: GRU, LSTM, and BiLSTM, and Transformer-based models: AraBERT, CAMeLBERT for Modern Standard Arabic, and XLM-RoBERTa. The experimental configuration involved a learning rate of 5e-6, batch size of 8 for training and 16 for testing, a maximum of 10 epochs, AdamW optimizer, and gradient accumulation steps of 8. TThe dataset was split into 80% training, 10% validation, and 20% testing. The result was that the Transformer-based models outperformed the RNN-based models in every task. In particular, the fine-tuned AraBERT model performed impressively well, with 100% accuracy for Question Particle Type classification, 94.95% accuracy for Intent classification, and 91.85% accuracy for Answer Type classification. CAMeLBERT and XLM-RoBERTa also performed impressively well, indicating the strength and versatility of the dataset across model architecture.

Apart from classification, the dataset was also integrated into a generative question-answering system with Alpaca + Gemma-9B Unsloth models. The system greatly benefited from the integration of the proposed dataset for conditioning the generation, outperforming baseline models by large margins. Specifically, the AAFAQ-augmented model yielded considerable improvements on BLEU (+37.6%), ROUGE-1 (+132.1%), ROUGE-2 (+129.8%), ROUGE-L (+132.1%), and BERTScore (+17.3%). Additionally, training time and memory usage were also optimized since the dataset was structured and attribute rich. A five-fold cross-validation also validated the dataset’s capacity to generalize, with stable and consistent performance across different data folds.

Although the dataset has broad coverage and consistency, some classes such as Sociology and Volunteering are less covered. Future updates will rectify this by obtaining additional questions in these classes through manual curation in order to have more balanced coverage across disciplines. Of broader importance, the impact of the dataset stretches into enhancing educational materials, enabling cognition-based competency tests, and benefiting multilingual research communities, particularly in language resource creation and information retrieval system development.

To build on what currently exists, future releases will have more sophisticated statistical analysis. Accompanying will be methods such as Chi-Square and Principal Component Analysis (PCA) for the analysis of inter-variable relationships and structural imbalances in the dataset. While the current version contains comprehensive frequency and correlation statistics—with items such as Question Particle and Intent highlighted—it will be more advanced methods like clustering and latent factor analysis that will be employed in the future. Also, we recognize the absence of demographic metadata, which may limit the potential for estimation of potential cultural or population-based bias. In the future, we will institute demographic-aware annotation standards and leverage bias analysis tools so that fairer and more inclusive dataset generation can be facilitated. All these will make future revisions of the dataset valid, robust, and responsive to shifting research requirements.

## Technical Validation

### Data cleaning

Following the initial aggregation, a rigorous data cleaning phase was undertaken. Approximately 1,500 questions were excluded based on predefined criteria: incomplete syntax, unresolvable ambiguities, irrelevance to general knowledge, high redundancy, or incoherence. Duplicate detection used string similarity and token-level matching algorithms. Step-by-step standardization consisted of orthographic normalization, tokenizing, and the exclusion of too vague questions and/or questions that did not map semantically to valid answers. Trained annotators uniformly applied these standards, with ambiguous instances being referred up for senior review.

### Data augmentation

Because the provided datasets only covered partially all the classes specified in our taxonomical proposal, we supplemented the dataset with data augmentation. This was done by downloading pertinent paragraphs from Arabic Wikipedia (https://ar.wikipedia.org) and manually crafting novel questions based on the obtained texts. Linguistic diversity was emphasized in the augmentation by including a wide range of question particles including “ما” (“what”), “لماذا” (“why”), “متى” (“when”), and “هل” (“yes/no” as in a question). Evidence questions were verified with care so that they were linguistically correct, semantically coherent, and structurally varied. Utilizing the content of Wikipedia conformed with the CC BY-SA 3.0 license policy, so derivative work could be done with correct referencing. This augmentation procedure helped stabilize the classes with sparse data coverage and enriched the dataset’s linguistic diversity and coverage.

### Taxonomy design

The dataset was developed using the AAFAQ framework, an extensible and component-based taxonomy for Arabic question classification. It was created by borrowing and building on top of other frameworks, such as the Li & Roth Question Taxonomy^1^, Bloom’s Taxonomy^28^, and the IQAS^29^. These models were reviewed and analyzed in light of the linguistic and cognitive complexity of Arabic, and their concepts were adapted to construct an Arabic-specific taxonomy that covers eleven dimensions of question classification. The AAFAQ taxonomy aims to capture both semantic and cognitive properties while considering the syntactic structures inherent in the Arabic language.

### Annotation and labeling process

The data was annotated by manual annotators based on a detailed guideline according to the AAFAQ Framework. All the questions were annotated with a total of eleven features: Question Particle, Question Particle Type, Question Type, List, Answer Type, Intent, Cognitive Level, Category, Subjectivity, Temporal Context, and Purpose Context. Prior to the actual annotation, the annotators were pre-trained using the annotation guidelines to make the annotation consistent and reliable. Detailed instructions were given, e.g., how to annotate the cognitive level in accordance with Bloom’s Taxonomy, how to deal with implicit questions, and how to distinguish between factoid and non-factoid questions. Subjectivity was determined based on whether the question asked for an opinion, judgment, or evaluative reasoning. Cognitive Level was annotated by matching the question objective with Bloom’s verbs translated and adapted to Arabic contexts (e.g., “ما الفرق بين…” (What is the difference between…) annotated as Analysis  (Give an example of…) annotated as Application (تطبيق)). Annotators received a training package containing a 30-page manual with examples, decision trees, and case studies. Ambiguities were resolved through consensus or adjudication by a lead annotator. Contextual blinding (e.g., hiding source identity) was used to mitigate annotator bias.

### Annotation consistency and inter-annotator agreement

For maintaining reliability and consistency in annotations, several rounds of validations were conducted. A random sample of the set comprising 10% were independently annotated by more than one annotator, and inter-annotator agreement (IAA) calculated using Cohen’s Kappa coefficient resulted in a score of 0.85. This reflects a strong degree of annotator consistency. Conflicts were addressed by consensus discussions that occurred each week as a way of improving the annotation guidelines. Overall, nearly 1,500 questions were discarded based on annotation conflict, non-relevance, duplications, or failing quality standards. Several rounds of quality assurance were applied throughout the process. The resulting dataset comprises 5,009 high-quality, consistently annotated entries across eleven classification dimensions and is compatible with generative systems such as Alpaca + Gemma-9B Unsloth^30^, reflecting strong internal validity.

### Licensing and ethical considerations

All data used in constructing the dataset was collected from openly available and license-compliant sources. The dataset does not include copyrighted or sensitive data. Content generated or adapted from Wikipedia fully complies with its Creative Commons Attribution-Share Alike 3.0 (CC BY-SA 3.0) license. In addition, a metadata file was prepared to record the original source for each question (e.g., DAWQAS, Wikipedia, WikiQAar, or XQUAD) to ensure full traceability.

## Supplementary information

Supplementary Table 1

## Data Availability

The custom code used for generating and processing the data in this study is available on GitHub at^31^.The repository includes the necessary scripts and dependencies required to reproduce the results.

## References

[1] Hamza, A., En-Nahnahi, N., Zidani, K. A. & El Alaoui Ouatik, S. An arabic question classification method based on new taxonomy and continuous distributed representation of words, *Journal of King Saud University - Computer and Information Sciences* **33**, 10.1016/j.jksuci.2019.01.001 (2021).

[2] Khedimi, S., Bouziane, A. & Bouchiha, D. Advancements and challenges in Arabic question answering systems: a comprehensive survey. *Brazilian Journal of Technology* **7**, e75604 (2024).

[3] Elfadil, S. S. A. N., Jarajreh, M. & Algarni, S. Question Answering Systems: A Systematic Literature Review, *International Journal of Advanced Computer Science and Applications*, **12**, 10.14569/IJACSA.2021.0120359 (2021).

[4] Essam, M. *et al*. Decoding Queries: An In-Depth Survey of Quality Techniques for Question Analysis in Arabic Question Answering Systems. *IEEE Access* **12**, 135241–135264, 10.1109/ACCESS.2024.3458466 (2024).

[5] Alwaneen, T. H., Azmi, A. M., Aboalsamh, H. A., Cambria, E. & Hussain, A. Arabic question answering system: a survey, *Artif Intell Rev*, **55**, 10.1007/s10462-021-10031-1 (2022).

[6] Alkhurayyif, Y. & Sait, A. R. W. A comprehensive survey of techniques for developing an Arabic question answering system, *PeerJ Comput Sci*, **9**, 10.7717/peerj-cs.1413 (2023). 10.7717/peerj-cs.1413 PMC10280590 37346617

[7] Biltawi, M. M., Tedmori, S. & Awajan, A. Arabic Question Answering Systems: Gap Analysis, *IEEE Access*, **9**, 10.1109/ACCESS.2021.3074950 (2021).

[8] Essam, M., Deif, M. A. & Elgohary, R. Deciphering Arabic question: a dedicated survey on Arabic question analysis methods, challenges, limitations and future pathways. *Artif Intell Rev* **57**, 251, 10.1007/s10462-024-10880-6 (2024).

[9] Li, X. & Roth, D. Learning question classifiers, 10.3115/1072228.1072378 (2002).

[10] Ahmed, W. & Anto P, B. Question Analysis for Arabic Question Answering Systems, *International Journal on Natural Language Computing*, **5**, 10.5121/ijnlc.2016.5603 (2016).

[11] Balla, H., Salvador,M. L. & Delany, S. J. Arabic Question Classification using Deep Learning, in *ACM International Conference Proceeding Series*, 10.1145/3562007.3562024 (2022).

[12] Alammary, A. S. Arabic Questions Classification Using Modified TF-IDF, *IEEE Access*, **9**, 10.1109/ACCESS.2021.3094115 (2021).

[13] Farghaly, A. & Shaalan, K. Arabic natural language processing: Challenges and solutions, *ACM Transactions on Asian Language Information Processing*, **8**, 10.1145/1644879.1644881 (2009).

[14] Alkhurayyif, Y. & Sait, A. R. W. Developing an Open Domain Arabic Question Answering System Using a Deep Learning Technique, *IEEE Access*, **11**, 10.1109/ACCESS.2023.3292190 (2023).

[15] Learning Question Classifiers. [Online]. Available: https://cogcomp.seas.upenn.edu/Data/QA/QC/ Accessed: Feb. 16 (2024)

[16] Ismail, W. S. & Homsi, M. N. DAWQAS: A Dataset for Arabic Why Question Answering System, in *Procedia Computer Science*, 10.1016/j.procs.2018.10.467 (2018).

[17] Yang, Y., Yih, W. T. & Meek, C. WIKIQA: A challenge dataset for open-domain question answering, *Conference Proceedings - EMNLP 2015: Conference on Empirical Methods in Natural Language Processing*, pp. 2013–2018, 10.18653/V1/D15-1237 (2015).

[18] Rajpurkar, P., Zhang, J., Lopyrev, K. & Liang, P. SQuad: 100,000+ questions for machine comprehension of text, *EMNLP 2016 - Conference on Empirical Methods in Natural Language Processing, Proceedings*, pp. 2383–2392, 10.18653/V1/D16-1264 (2016).

[19] Malhas, R., Mansour, W. & Elsayed, T. Qur’an QA 2022: Overview of The First Shared Task on Question Answering over the Holy Qur’an, in *5th Workshop Open-Source Arabic Corpora and Processing Tools with Shared Tasks on Qur’an QA and Fine-Grained Hate Speech Detection, OSACT 2022 - Proceedings at Language Resources and Evaluation Conference, LREC*, (2022).

[20] Al-Omari, H. & Duwairi, R. So2al-wa-Gwab: A New Arabic Question-Answering Dataset Trained on Answer Extraction Models, *ACM Transactions on Asian and Low-Resource Language Information Processing*, **22**, 10.1145/3605550 (2023).

[21] masun/DAWQAS: A Dataset for Arabic Why Question Answering System. [Online]. Available: https://github.com/masun/DAWQAS Accessed: Mar. 28 (2025).

[22] wiki_qa_ar · Datasets at Hugging Face. [Online]. Available: https://huggingface.co/datasets/wiki_qa_ar Accessed: Feb. 15 (2024).

[23] xquad/xquad.ar.json at master · google-deepmind/xquad. [Online]. Available: https://github.com/google-deepmind/xquad/blob/master/xquad.ar.json Accessed: Feb. 15 (2024).

[24] Task-B/data · main · bigIR/Quran QA 2023 · GitLab. [Online]. Available: https://gitlab.com/bigirqu/quran-qa-2023/-/tree/main/Task-B/data?ref_type=heads Accessed: Feb. 15 (2024).

[25] Task-A/data · main · bigIR/Quran QA 2023 · GitLab. [Online]. Available: https://gitlab.com/bigirqu/quran-qa-2023/-/tree/main/Task-A/data Accessed: Feb. 15 (2024).

[26] Essam, M., Deif, M., Algamdi, S. A. & Elgohary, R. A benchmark Arabic dataset for question classification with AAFAQ taxonomy [Dataset]. *Dryad.*10.5061/dryad.9w0vt4brx (2025).

[27] Cramér, H. *Mathematical methods of statistics*, vol.9, Princeton university press (1999).

[28] Blyth, W. A. L., Bloom, B. S. & Krathwohl, D. R. Taxonomy of Educational Objectives. Handbook I: Cognitive Domain, *British Journal of Educational Studies*, **14**, 10.2307/3119730 (1966).

[29] Neji, Z., Ellouze, M. & Belguith, L. H. IQAS: Inference question answering system for handling temporal inference, in *Proceedings of the 2016 International Symposium on INnovations in Intelligent SysTems and Applications, INISTA*. 10.1109/INISTA.2016.7571832 (2016).

[30] GitHub - unslothai/unsloth: Finetune Llama 3.2, Mistral, Phi & Gemma LLMs 2-5x faster with 80% less memory. [Online]. Available: https://github.com/unslothai/unsloth Accessed: Oct. 14 (2024).

[31] Abdelaziz, M. E., Deif, M. A., Algamdi, S. A. & Elgohary, R. AAFAQ Dataset - Arabic Question Classification code. [Online]. Available: https://github.com/Mohanad-Deif/AAFAQ-Dataset Accessed: Jun. 17 (2025). 10.1038/s41597-025-05688-0 PMC12361461 40825807

