# Supplementary material for: A Benchmark Arabic Dataset for Arabic Question Classification using AAFAQ Framework
Source: Sci Data. 2025 Aug 18;12:1444. doi: 10.1038/s41597-025-05688-0 (PMC12361461; doi:10.1038/s41597-025-05688-0)
Supplement: Supplementary file 1 — Supplementary Table 1 [file 41597_2025_5688_MOESM1_ESM.doc]

**Supplementary Table [1]** : Overview of The Dataset Features, Types, and Categories.

| **Feature** | **Type** | **Categories/Values** | **N of Categories** | **Description** |
| --- | --- | --- | --- | --- |
| **QuestionID** | Numerical | Integer | _ | Unique identifier for each question. |
| **QuestionText** | Text | Text | _ | The text of the question itself. |
| **QuestionParticle (أداة السؤال)** | Categorical | "كيف" (How), "من" (Who), "أين" (Where), "لم" (Why), "لماذا" (Why), "متى" (When), "أيان" (When), "كم" (How many, How much), "ما" (What), "أي" (Which), "هل" (Yes/No), Imperative forms (e.g., Write, Mention, Clarify, etc.), "ضمني" (Implicit) | 20+ | The tool or word used to ask the question, including interrogatives, boolean, imperative, and implicit forms. |
| **QuestionParticleType (نوع أداة الاستفهام)** | Categorical | "أداة استفهام" (Interrogative particle), "فعل أمر" (Imperative verb), "ضمني" (Implicit) | 3 | Specifies whether the question uses an interrogative particle, imperative verb, or is implicit. |
| **QuestionType (نوع السؤال)** | Categorical | "واقعي" (Factoid), "غير واقعي" (Non-Factoid) | 2 | Defines whether the question is based on factual information or not. |
| **List (قائمة)** | Categorical | True, False | 2 | Indicates if the answer is a list. |
| **AnswerType (نوع الإجابة)** | Categorical | "وصف" (Description), "رقم" (Number), "تاريخ" (Date), "نعم/لا" (Boolean), "مكان" (Place), "عاقل" (Human), "غير عاقل" (Object), "كيان" (Entity), "كمية" (Quantity), "ظرف زمان" (Time Adverbial), "سبب" (Reason) | 12 | Specifies the expected type of answer, such as text, number, date, location, or entity. |
| **Intent (النية)** | Categorical | "معلوماتي" (Informational), "رأي" (Opinion), "اختيار" (Selection), "مقارنة" (Comparison), "تعريف" (Definition), "شرح" (Explanation), "استدلال" (Inference), "حساب" (Calculation), "اقتراح" (Suggestion), "ترتيب" (Ordering), Explanation - "تفسير", Prediction - "تنبؤ"  Parse – "إعراب",  Planning – "تخطيط". | 14 | Represents the intent or purpose of the question. |
| **CognitiveLevel (المستوى المعرفي)** | Categorical | "معرفة" (Knowledge), "فهم" (Comprehension), "تطبيق" (Application), "تحليل" (Analysis), "تركيب" (Synthesis), "تقييم" (Evaluation) | 6 | The cognitive skill level required to answer the question. |
| **Category (الفئة)** | Categorical | "الثقافة" (Culture), "العلوم" (Science), "التعليم" (Education), "الصحة" (Health), "البيئة والطاقة" (Environment and Energy), "التكنولوجيا" (Technology), "التاريخ" (History), "الاقتصاد والعمل" (Economy and Work), "الجغرافيا" (Geography), "السياسة والقانون" (Politics and Law), "الدين" (Religion), "الرياضة" (Sports), "البيولوجيا" (Biology), "الترفيه" (Entertainment), "السفر والسياحة" (Travel and Tourism), "التطوع" (Volunteering), "علم الاجتماع" (Sociology) | 17 | Domain or field of the question, covering a wide variety of topics. |
| **Subjectivity (الذاتية)** | Categorical | "ذاتي" (Subjective), "موضوعي" (Objective) | 2 | Indicates if the question is subjective or objective in nature. |
| **TemporalContext (السياق الزمني)** | Categorical | "غير متعلق بالوقت" (Timeless), "ماضي" (Past), "حاضر" (Present), "مستقبل" (Future), "وقت محدد" (Specific Time) | 5 | Defines the time frame the question is concerned with. |
| **PurposeContext (سياق الغرض)** | Categorical | "جمع المعلومات" (Information Gathering), "حل المشكلات" (Problem Solving), "اتخاذ القرارات" (Decision Making), "التفاعل الاجتماعي" (Social Interaction), "تنبؤ" (Prediction) | 5 | The purpose or goal of asking the question. |
| **AnswerSourceText (نص مصدر الإجابة)** | Text | Text | _ | The text from which the answer is derived |
| **Answer** | Text | Text | _ | The actual answer to the question |
